# Pilot Retrospective Evaluation of a Balancing and Optimizing Injection Pattern for the Frontalis Muscle Using LetibotulinumtoxinA

**DOI:** 10.3390/toxins17120594

**Published:** 2025-12-11

**Authors:** Konstantin Frank, Lukas Prantl, Vanessa Brebant, Syed Haq

**Affiliations:** 1 Department of Plastic, Hand and Reconstructive Surgery, University Hospital Regensburg, 93053 Regensburg, Germany; lprantl@csj.de (L.P.);; 2 Haq Medical Consultancy and AM Aesthetics, 10 Harley St, London W1G 9PF, UK

**Keywords:** LEBO, neuromodulator, forehead injection, frontalis, forehead lines

## Abstract

Signs of aging in the upper face arise from multimodal changes in facial anatomy, contributing to concerns such as eyebrow ptosis and forehead lines. While neurotoxin injections are widely used to address these lines, the anatomical variability of the frontalis muscle presents procedural challenges. This retrospective analysis aimed to introduce and preliminarily evaluate a structured injection pattern for forehead treatment, developed with attention to the biomechanics of upper facial musculature. A total of 24 patients (mean age 42.5 ± 9.1 years) treated with a standardized injection scheme using letibotulinumtoxinA were included. All subjects also received concomitant glabellar treatment. The protocol incorporated identification of the line of convergence and targeted injections at defined points to balance elevation, optimize muscular activity, and minimize the risk of eyebrow descent. Forehead line severity was assessed at rest and during animation, and three-dimensional surface imaging was used to quantify vertical skin displacement. At baseline, 79.2% of patients presented with severe dynamic forehead lines, and 29.1% exhibited severe static lines. After two weeks, 62.5% showed no dynamic lines and 41.7% showed no static lines. All subjects demonstrated a ≥1-point improvement in dynamic line severity, with 87.5% achieving a ≥2-point improvement. For static lines, 95.8% achieved a ≥1-point improvement and 20.8% showed a ≥2-point improvement after two weeks. The mean dosage was 17.8 ± 0.7 U. Two patients (8.3%) required a touch-up, and no adverse events were observed. These findings suggest that this structured injection approach may offer a consistent method for addressing forehead lines; however, the results should be interpreted within the limitations of a small, uncontrolled retrospective series. Prospective controlled studies with larger populations are needed to further validate the technique.

## 1. Introduction

Signs of aging in the upper face arise from multimodal changes in facial anatomy. Common concerns leading patients to seek rejuvenation include temporal concavity, eyebrow ptosis, blepharochalasis and the formation of static glabellar and horizontal forehead lines [1,2,3,4,5,6,7,8]. Dynamic and static forehead lines are not exclusive to elderly patients but can also be bothersome to younger individuals, with treatment typically involving neurotoxin injections to ameliorate their appearance at rest and upon contraction, i.e., elevation of the eyebrow [7,9].

Unlike the treatment of glabellar lines, where the relatively predictable anatomy of the corrugator and procerus muscles guides the procedure, or lateral canthal lines, dictated by the superficial nature and clear topographically detectable extent of the orbicularis oculi muscle, addressing forehead lines necessitates a more comprehensive understanding of the anatomy and biomechanics of the upper facial musculature. This complexity is primarily due to the frontalis muscle’s bi-directional contraction pattern, which creates a variable line of convergence [10]. Identifying this line on an individual basis is crucial, as injections below it can and most commonly will cause eyebrow depression. The frontalis muscle’s elevating fibers are the only ones capable of raising the eyebrow, counteracting the depressor muscles, including the corrugator, procerus, and orbicularis oculi. Additionally, the decussation pattern of the frontalis muscle varies significantly causing the morphology of the forehead lines to vary significantly [11]. This pattern is influenced by the size of the aponeurosis connecting the two frontalis muscle bellies: a larger aponeurosis results in more laterally oriented fibers, clinically observed as wavy forehead lines, while a smaller aponeurosis leads to straight forehead lines. The current labeling for botulinumtoxinA recommends a dose of 20 Units divided across five injection sites for the treatment of forehead lines. While this guidance provides a simple and standardized protocol, it does not adequately account for the complex and highly variable anatomy of the forehead.

This approach assumes uniform anatomy and biomechanics across all patients, ignoring key individual variations.

Moreover, the variability in botulinum neurotoxins (BoNTs), including their non-interchangeability due to distinct manufacturing processes, further complicates treatment protocols. Each BoNT product, owing to unique characteristics such as serotype and formulation, interacts differently with tissue microenvironments, necessitating tailored injection schemes particularly in challenging areas like the forehead. This highlights the need for a specific and appropriate injection pattern, such as the one introduced in this investigation, to optimize patient outcomes.

Several individualized and algorithmic injection concepts have recently been proposed to move beyond fixed “safe zone” or grid-based forehead protocols. The One21 technique, for example, uses a detailed 21-point mapping of the upper face with incobotulinumtoxinA, allowing dose and injection sites to be tailored to each patient’s specific contraction pattern, eyebrow position, and degree of muscle hyperactivity [12]. Similarly, consensus recommendations for tailored botulinum toxin type A injections in the forehead advocate a “zonal” or “mapped” approach that accounts for individual facial anatomy, muscle tone (kinetic, hyperkinetic, hypertonic), and lateral frontalis activity, rather than applying a uniform injection template. Global and regional esthetic consensus statements further emphasize an integrated, patient-specific treatment plan in which neuromodulation is adjusted according to muscle mass, vector of pull, and esthetic goals, moving away from rigid standard patterns [13,14,15]. Within this evolving landscape, the LEBO (Lateralization at the right Elevation, Balancing and Optimization) algorithm is intended as a structured yet pragmatic framework that explicitly incorporates the line of convergence and defines dedicated balancing and optimization points to harmonize brow position and forehead movement. Unlike many previously described schemes that are either purely product-agnostic or designed around fixed point maps, LEBO focuses on dynamic assessment of individual frontalis biomechanics and lateralization patterns, providing a stepwise, anatomy-guided approach that can be readily adapted to different clinical presentations.

Clinically, injecting the frontalis muscle poses challenges, especially for first-time patients or less experienced physicians. Concerns include the risk of creating eyebrow ptosis, incorrect injection sites leading to unsatisfactory results and frequent touch-ups, and esthetically unpleasing outcomes such as “spock brow”, asymmetric brow elevation or hyperactive cranial aspects of the frontalis muscle. To enhance patient satisfaction, safety and reduce touch-up frequency, and optimize dosage requirements, a new injection pattern has been introduced. This investigation aims to introduce this injection pattern and retrospectively assess its efficacy based on chart reviews and three-dimensional surface imaging analysis.

## 2. Results

### 2.1. Overall Findings

A total of 24 subjects were treated with the novel introduced injection pattern. These patients had a mean age of 42.5 ± 9.1 years [Range: 27–66 years] and consisted of 10 males (41.7%) and 14 females (58.3%) (Table 1). At baseline, a total of 5 subjects (20.8%) presented with moderate dynamic forehead lines, while 19 subjects (79.2%) presented with severe dynamic forehead lines. One subject (4.2%) presented with no static forehead lines, nine subjects (37.5%) with mild static forehead lines and seven subjects (29.1%) with moderate and severe static forehead lines each. The mean dosage used for treatment of the frontalis muscle was 17.8 ± 0.7 U. All subjects received six injection points at the level of the C–line and the bilateral vertical intersection of the lateral eyebrow, apex and medial eyebrow (12U in total). Two balancing points (one on each side) were injected in 100% of the subjects with 1U each, yielding a total of 2U. The four optimization points were injected in 100% of the subjects. The mean dosage for the optimization point was 3.75 ± 0.7 U. 2 patients (8.3%) required a touch—up after 2 weeks. No adverse reactions were reported after 2 weeks.

### 2.2. Static and Dynamic Forehead Line Severity

At follow-up, 15 patients (62.5%; 95% CI 42.7–78.8) presented with no dynamic forehead lines, while 9 patients (37.5%; 95% CI 21.2–57.3) presented with mild dynamic forehead lines. A total of 10 patients (41.7%; 95% CI 24.5–61.2) presented with no static forehead lines, while 12 (50.0%; 95% CI 31.4–68.6) presented with mild static forehead lines and 2 (8.3%; 95% CI 2.3–25.8) with moderate static forehead lines. Overall, 100% of the subjects (24/24; 95% CI 86.2–100.0) showed a ≥1-point improvement in dynamic forehead lines, while 21/24 patients (87.5%; 95% CI 69.0–95.7) showed a ≥2-point improvement in their dynamic forehead lines. For static forehead lines, 23/24 patients (95.8%; 95% CI 79.8–99.3) showed a ≥1-point improvement after 2 weeks, while 5/24 subjects (20.8%; 95% CI 9.2–40.5) showed a ≥2-point improvement.

### 2.3. Skin Displacement

Vertical movement at baseline upon maximum contraction of the frontalis muscle was 4.10 ± 0.5 mm, while it was 2.02 ± 0.6 mm after 2 weeks, with *p* < 0.001.

## 3. Discussion

The anatomy of the frontalis muscle is notably variable among individuals, significantly influencing the clinical results of forehead line treatments. This variability primarily originates from the different patterns of muscle fiber decussation and the size of the aponeurosis connecting the two frontalis muscle bellies [2,3,4,10,11]. A larger aponeurosis tends to result in more laterally oriented muscle fibers, producing wavy forehead lines, while a smaller aponeurosis leads to straight lines. This anatomical variation necessitates a highly individualized approach to the treatment of forehead lines, as the line of convergence—where the frontalis muscle’s bi-directional contraction pattern meets—can significantly impact the outcome [10].

Understanding the line of convergence is crucial for achieving optimal results and preventing complications such as eyebrow ptosis. The line of convergence can be clinically identified by having the patient elevate their eyebrow while gently placing an index finger on the forehead to detect the point where the finger is neither pushed cranially nor caudally. Injections placed below this line can often lead to undesirable eyebrow depression, as these points might inhibit the elevating action of the frontalis muscle’s fibers. Hence, precise identification and consideration of the line of convergence are essential to avoid such adverse effects.

The delicate nature of the frontalis muscle anatomy presents a narrow therapeutic window, making it challenging to achieve the desired esthetic outcomes without causing complications. This complexity is compounded by varying patient requests, as some patients prefer a natural look with retained forehead movement, while others opt for a “frozen” look with no muscle activity. Balancing these preferences requires a nuanced understanding of the muscle’s function and careful injection techniques [15].

The newly introduced injection scheme aims to address these challenges by providing a structured and easy-to-follow approach. This scheme incorporates six injection points from medial to lateral at the correct height, identified by the acronym LEBO (Lateralization, Elevation, Balancing points, Optimization points).

Lateralization and Elevation (LE): These points ensure that the muscle fibers responsible for elevating the eyebrow are avoided as much as possible by injecting at the level of the line of convergence, preventing ptosis and achieving a balanced eyebrow positioning.

Balancing points (B): These points are injected superficially to counteract hyperactive lateral pull and avoid the Mephisto sign.

Optimization points (O): These points are identified based on individual areas of high muscular activity and are injected deeply to fine-tune the treatment.

In this small, uncontrolled cohort, the majority of subjects presented at baseline with severe dynamic forehead lines and varying degrees of static lines. Following treatment with the LEBO-based injection pattern in combination with standard glabellar treatment, a significant reduction in both dynamic and static forehead lines was observed. All patients demonstrated at least a one-point improvement in line severity. These findings are hypothesis-generating and suggest that a structured algorithm such as LEBO may facilitate consistent esthetic outcomes; however, in the absence of a comparator arm and given the concomitant treatment of the glabella, the observed improvements cannot be attributed to the LEBO technique alone.

The LEBO technique was designed as an individualized approach that tailors injection patterns based on anatomical and functional characteristics rather than predetermined sex-based dosing. While it is well established that men typically have greater muscle mass and may require higher doses of botulinum toxin, the LEBO method inherently accounts for these differences through its structured yet flexible injection strategy, focusing on real-time assessment of muscle activity and forehead morphology.

In this study, both male and female patients received the same base dose according to the LEBO protocol, which prioritizes muscle activity mapping over sex-based dosing. This is in line with contemporary trends toward individualized, pattern-based dosing, in which sex is only one of several parameters considered in clinical decision-making.

Apart from assessing effectiveness using validated scales, objective measurements of forehead movement were utilized. The findings indicate a significant reduction in vertical movement of the frontalis muscle post-treatment. Initially, the vertical movement at baseline upon maximum contraction of the frontalis muscle was 4.10 ± 0.5 mm. After two weeks, this movement decreased to 2.02 ± 0.6 mm, with a highly significant *p*-value of <0.001. This reduction in movement highlights the impact of treatment on limiting the activity of the frontalis muscle, which is essential in achieving the desired esthetic outcome. The substantial decrease in movement suggests that the frontalis muscle was significantly weakened, leading to less elevation of the eyebrow and a smoother forehead appearance. Within the limitations of the study design, this objective reduction underscores the potential of a structured algorithm to minimize excessive dynamic activity while preserving balanced upper-face function.

The mean dosage used was 17.8 ± 0.7 U, with a consistent injection strategy across patients. The comprehensibility and reproducibility of the LEBO injection scheme, combined with its adaptability to individual anatomical variations, may support its use as a practical tool for clinicians aiming to enhance patient satisfaction and minimize the need for touch-ups in routine practice. Furthermore, no adverse drug reactions were reported, indicating that this approach appears safe when performed correctly.

The LEBO technique stands out in its ability to address the anatomical and functional variability of the forehead, offering a more tailored and precise approach compared to some other commonly used techniques. The “Safe Zone” technique involves 6–10 evenly spaced injection points distributed in 1–2 horizontal lines, each placed at least 1.5–2 cm above the orbital rim to minimize the risk of brow ptosis. While effective in avoiding ptosis, this method lacks the individualized approach of LEBO, as it does not explicitly take into account patient-specific muscular activity or the line of convergence.

The V-shape technique, which involves 4–6 injection points forming a V-shape in women or a straight line in men, considers gender-specific esthetic patterns but primarily focuses on appearance without fully addressing the underlying muscle biomechanics as the LEBO technique does. Furthermore, the V-shape technique typically uses a higher total dose of 20–60 units, which contrasts with the more conservative yet apparently effective dosing of the LEBO approach in our cohort.

The 2-step technique treats the upper and lower portions of the frontalis muscle in two separate sessions, with follow-up to adjust brow positions. While this method allows for fine-tuning, it introduces delays in achieving the final results. In contrast, the LEBO technique aims to provide balanced outcomes within a single session, which may be more efficient from a practical standpoint, although direct comparative data are currently lacking.

The Microbotox technique delivers diluted botulinum toxin in microdroplets across the entire forehead, targeting fine lines and wrinkles. While it is effective for addressing certain wrinkle patterns, such as Type III (mild dynamic wrinkles) and Type IV (moderate/severe wrinkles), Microbotox lacks the precision of the LEBO technique. Its broad application often overlooks critical individualized factors such as the line of convergence and areas of hyperactive muscle activity, which are central to the LEBO approach. The LEBO technique inherently allows for customization to achieve varying degrees of frontalis muscle paralysis based on patient preferences and clinical goals. This flexibility is achieved by adjusting the number, placement, and dosage of optimization points during the treatment. For patients desiring a natural appearance with minimal muscle relaxation and preserved facial expression, fewer optimization points are selected and lower dosages are used. Conversely, for patients seeking more pronounced paralysis to reduce the appearance of dynamic forehead lines, additional optimization points can be incorporated and higher dosages may be administered.

The recently published consensus recommendations by Choi et al. provide comprehensive guidelines for the treatment of the upper face with LetibotulinumtoxinA, focusing on standardized injection techniques and dosing recommendations [16]. These recommendations were developed by an expert panel to provide guidance on best practices for forehead, glabellar, and lateral canthal line treatments.

Compared to the LEBO technique presented in this study, the consensus recommendations emphasize a structured, predefined approach to forehead injections, taking into account forehead height, standardized injection points, and recommended dosages. The expert panel recommends a total dose of 14 units for forehead injections, using 2 units per point at seven injection sites. In contrast, the LEBO technique incorporates a more individualized approach by considering key anatomical and functional variables such as the natural brow shape, the line of convergence, and the lateral pull of the eyebrow. This results in a tailored injection pattern where the total dose ranges between 16 and 18 units, depending on patient-specific muscle activity.

Beyond these consensus recommendations, several recently proposed individualized algorithms and consensus-based tailored approaches for upper-face BoNT injection (such as the One21 concept, LADs-based designs, and related expert-driven schemas) similarly emphasize mapping of individual muscle activity, forehead height, and line patterns to guide dosing and injection point selection. LEBO can be regarded as one such structured algorithm, specifically optimized for the forehead with an explicit focus on the line of convergence and lateral pull. The present data should therefore be interpreted as contributing to this broader movement toward individualized, algorithm-based treatment rather than as establishing the superiority of one specific technique over others. Head-to-head comparative studies between LEBO and these newer individualized approaches will be required to define their relative benefits and optimal indications more clearly.

Additionally, the consensus recommendations emphasize the importance of gender-specific considerations, noting that men generally have larger and more active musculature, which may require different dosing strategies. In contrast, the LEBO approach does not differentiate between male and female patients in terms of pre-defined dosing but instead relies on real-time assessment of muscle activity to guide injections. The results of the present study indicate that the LEBO technique is effective for both male and female patients within the observed cohort, demonstrating significant reductions in dynamic and static forehead lines. Furthermore, the balancing points and lateralizing points in the LEBO algorithm can also be modified to fine-tune the overall result. By carefully assessing the patient’s anatomy and functional muscle activity during the pre-treatment evaluation, the injector can tailor the approach to ensure that the desired degree of relaxation is achieved without compromising the natural movement or esthetic balance of the forehead. This adaptability ensures that the LEBO technique can cater to a broad spectrum of patient needs, from subtle enhancements to more pronounced reductions in dynamic and static forehead lines.

The unique characteristics of different BoNT products, including their serotypes and formulations, further necessitate customized approaches. These variations affect how the toxin interacts with the tissue microenvironment, particularly in complex areas like the forehead, where precise and individualized injection patterns are essential. The LEBO scheme’s structured approach, focusing on Lateralization, Elevation, Balancing, and Optimization points, offers a solution by considering these variations, aiming for effective treatment while minimizing complications such as eyebrow ptosis. The significant reduction in both dynamic and static forehead lines observed in this study underscores the potential usefulness of such a scheme, while also highlighting the need for further confirmatory studies.

**Figure 1:**
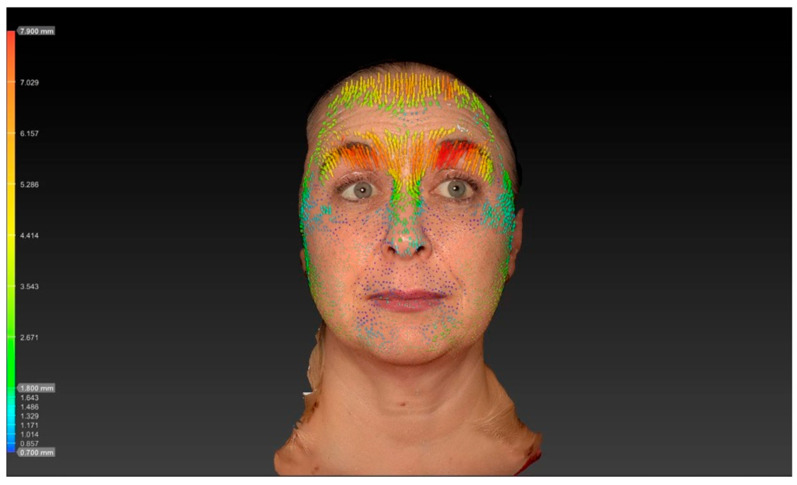
Vector analysis reveals pronounced vertical skin displacement of the forehead during maximal frontalis contraction, indicated by dense yellow and red vectors.

**Figure 2:**
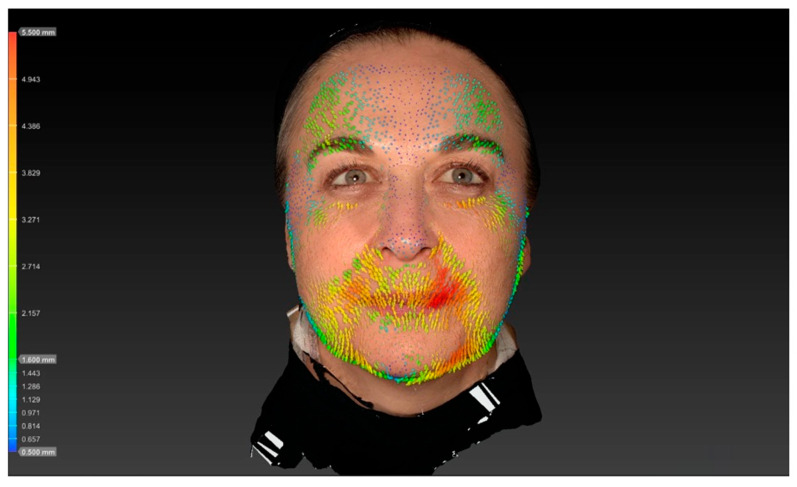
Two weeks after treatment: markedly reduced vector length and magnitude are visible, reflecting diminished frontalis muscle activity, with mean vertical displacement decreasing to 2.02 ± 0.6 mm (*p* < 0.001). These 3D surface vector maps objectively illustrate the functional reduction in forehead movement following botulinum toxin injection and demonstrate the efficacy of the LEBO injection scheme in achieving balanced muscle modulation.

The reduction in vertical forehead movement following botulinum toxin treatment in this study was quantified using 3D surface imaging, demonstrating a decrease from 4.10 ± 0.5 mm at baseline to 2.02 ± 0.6 mm after two weeks (*p* < 0.001). This objective measurement provides insight into the functional impact of the LEBO technique on frontalis muscle contraction. Previous work, including that by Kwon et al., has employed 3D surface imaging to analyze displacement patterns of the frontalis muscle after BoNT injection. However, these studies primarily reported global displacement fields rather than focusing specifically on quantified vertical movement amplitudes in the context of a predefined injection algorithm such as LEBO. Our analysis, therefore, adds complementary data by quantifying vertical movement reduction associated with an anatomically guided forehead injection scheme. Direct comparisons with other injection techniques or different toxin formulations assessed with the same 3D methodology are not yet available, and future studies using standardized imaging protocols will be important to enable such comparisons (Figure 1 and Figure 2).

This study has several limitations. First, the sample size of only 24 subjects is relatively small, which limits the generalizability of the findings. A larger, more diverse population would be necessary to validate these results further. Second, only one type of botulinum toxin at a specific reconstitution (4 U/0.1 mL) was used, which may not be directly applicable to other formulations or concentrations. Future studies utilizing varying toxin types and concentrations could provide additional insights. Third, the simultaneous injection of the glabella region introduces a confounding variable, as the observed outcomes may not exclusively reflect the efficacy of the LEBO technique on the frontalis muscle. The interaction between the treatments in these regions could influence the overall results, and further research isolating the effects of the LEBO technique would be beneficial. Moreover, the lack of a comparator arm and absence of randomization preclude any conclusions about relative efficacy versus other established injection patterns; the present findings should therefore be interpreted as reflecting the effect of a combined upper-face treatment protocol in which LEBO was used for the forehead, rather than proof of superiority of LEBO per se. Additionally, the study had a relatively short follow-up period, which limits our ability to assess the long-term efficacy and durability of the results. Longer follow-up studies are necessary to determine whether the initial outcomes are sustained over time. Finally, while the study design focused on a tailored approach, the lack of direct comparisons to other techniques in a controlled setting limits the ability to conclusively establish the comparative performance of the LEBO technique.

One notable strength of the study is the use of 3D vectors and standardized scales, which help in accurately assessing the injection technique. This methodological rigor supports the reliability of the findings and their potential reproducibility in clinical practice.

The low touch-up rate of approximately 8–9% with this technique is another noteworthy observation. Touch-ups can be bothersome for both patients and physicians, as they often require additional appointments and can increase the complaint rate. By minimizing the need for touch-ups, an algorithmic scheme such as LEBO may enhance patient satisfaction and reduce the burden on healthcare providers. However, given the small sample size, single-center setting, and short follow-up, these data should be interpreted with caution and cannot, at this stage, justify changes to standard follow-up protocols. In line with current best practice and medicolegal prudence, a structured post-treatment assessment, including a reassessment around two weeks when appropriate, remains advisable and should be discussed with patients as part of shared decision-making.

The narrow therapeutic window of frontalis injection due to high anatomical variability remains a critical challenge in forehead line treatments. The frontalis aponeurosis exhibits high variance in its cranio-caudal extent, lateral extent, and cranio-caudal positioning, resulting in different forehead line patterns. Some patients have straight lines, while others have wavy lines, depending on these anatomical factors. Finding the right balance between overtreating and undertreating is challenging and often results from misjudging the anatomy and treating it incorrectly. The LEBO scheme addresses this issue by providing a structured approach that explicitly considers these anatomical variations and tailors the treatment to individual needs.

This study has several limitations. First, the sample size of only 24 subjects is relatively small, which limits the generalizability of the findings. A larger, more diverse population would be necessary to validate these results further. Second, only one type of botulinum toxin at a specific reconstitution (4U/0.1 mL) was used, which may not be directly applicable to other formulations or concentrations. Future studies utilizing varying toxin types and concentrations could provide additional insights. Third, the simultaneous injection of the glabella region introduces a confounding variable, as the observed outcomes may not exclusively reflect the efficacy of the LEBO technique on the frontalis muscle. The interaction between the treatments in these regions could influence the overall results, and further research isolating the effects of the LEBO technique would be beneficial. Additionally, the study had a relatively short follow-up period, which limits our ability to assess the long-term efficacy and durability of the results. Longer follow-up studies are necessary to determine whether the initial outcomes are sustained over time. Finally, while the study design focused on a tailored approach, the lack of direct comparisons to other techniques in a controlled setting limits the ability to conclusively establish the superiority of the LEBO technique.

The low touch-up rate of 8.3% with this technique is another significant advantage of the presented technique, or rather, algorithm. Touch-ups can be bothersome for both patients and physicians, as they often require additional appointments and can increase the complaint rate. By minimizing the need for touch-ups, the LEBO scheme not only enhances patient satisfaction but also reduces the burden on healthcare providers. Due to the low 9.1% touch-up rate, it could potentially be agreed with the patient that no further standardized re-assessment appointments after 2 weeks are necessary.

## 4. Conclusions

The present retrospective analysis suggests that the LEBO injection scheme may facilitate a structured and individualized approach to the treatment of forehead lines, taking into account key anatomical and functional variables such as the line of convergence and lateral brow pull. Within a cohort of 24 patients treated with a standardized LetibotulinumtoxinA protocol for both the forehead and glabella, significant short-term improvements in dynamic and static forehead line scores were observed, accompanied by a low touch-up rate and an absence of serious adverse events.

However, these findings must be interpreted in light of several important limitations, including the retrospective, single-center design, the small sample size, the lack of a control or comparator group, the simultaneous glabellar injection as a potential confounder, and the relatively short 2-week follow-up period. As such, the current data should be considered hypothesis-generating rather than definitive evidence of superiority or generalizability.

Taken together, LEBO may be regarded as a promising, reproducible, and adaptable algorithmic framework for forehead treatment within the broader movement toward individualized upper-face BoNT injection strategies. Future prospective, randomized, and adequately powered studies with longer follow-up and direct comparison to other established algorithms and consensus-based approaches are required to confirm these preliminary observations, to clarify the specific contribution of LEBO relative to concomitant treatments, and to define its optimal role in clinical practice.

## 5. Materials and Methods

### 5.1. Study Sample

The patients were treated in the outpatient department of the Caritas St. Josef Hospital Regensburg and received treatment of the frontalis and glabellar lines. According to the physician’s standardized treatment protocol, the dosage was recorded and a photo of the patient at baseline and a standard 2–week follow-up was taken. The data of the patients was retrospectively analyzed. Prior to the analysis, a positive ethical votum for the retrospective analysis was received. Contraindications for BTX treatment included hypersensitivity to botulinum toxin type A or any of its excipients, active infections at or near the injection sites, and known neuromuscular disorders such as myasthenia gravis, Eaton-Lambert syndrome, or amyotrophic lateral sclerosis. Additionally, patients who were pregnant or breastfeeding, or those with a history of adverse reactions or complications from previous BTX treatments, were excluded from the study. These exclusions ensured that only patients deemed clinically appropriate for standard BTX treatment were included in the analysis. The principles of the Declaration of Helsinki and the applicable sections of the respective national laws were adhered to. The retrospective analysis included a review of the patient information at baseline and 2 weeks after initial treatment. The retrospective analysis included patients between January 2024 and November 2024.

### 5.2. Treatment Algorithm

The injection scheme was designed to account for individual upper-face anatomy and incorporates the following elements: natural brow shape, the line of convergence, lateral pull of the eyebrow, and areas of hyperactive muscular activity in the frontalis muscle.

The first step of the injection scheme is identification of the line of convergence. This is clinically determined by asking the patient to maximally elevate the eyebrows while the injector gently places an index finger without pressure on the central forehead. The vertical level at which the finger is neither pushed cranially nor caudally during contraction is defined as the line of convergence.

Once the line of convergence is identified, three injection points are marked bilaterally at this level. These points are located at the imaginary vertical intersections through the medial eyebrow, the apex of the eyebrow, and the lateral eyebrow. Each of these six points is injected intramuscularly (deep) with 2 units of letibotulinumtoxinA.

Next, the balancing point is determined. The strongest vector of frontalis contraction is identified upon eyebrow elevation, and an imaginary line is drawn between the nasal ala and this vector. Along this line, approximately 1.5–2.0 cm cranial to the eyebrow, a single balancing point is injected with 1 unit administered superficially. This aims to mitigate excessive lateral pull of the eyebrow and reduce the risk of a Mephisto sign.

The final, individualized component of the scheme consists of optimization points, defined as areas of particularly high muscular activity in the frontalis. These areas are identified by palpation and observation during maximal contraction and are injected with 1 unit each, intramuscularly (deep), at up to a maximum of four points.

In summary, the lateralizing points at the level of the line of convergence—where cranial and caudal vectors of frontalis movement meet—are injected with 2 units each deep intramuscularly, the balancing point is injected superficially with 1 unit, and up to four optimization points are injected deep with 1 unit each. This results in a total frontalis dose of 16–18 units of letibotulinumtoxinA per subject, depending on the number of optimization points required.

All patients also received treatment of the glabellar complex using a standard 5-point injection scheme, which is a widely recognized and clinically established approach for glabellar lines. This technique targets the primary muscles responsible for glabellar frown lines, including the corrugator supercilii and the procerus.

Five anatomical points were marked as follows: two symmetrical injection points were placed over the medial aspects of the corrugator supercilii muscles, approximately 1 cm above the medial canthus of each eye, targeting the medial fibers responsible for vertical frown lines. Two additional points were positioned laterally, approximately 1 cm from the medial corrugator points, to treat the lateral portions of the corrugator supercilii. The fifth point was injected into the belly of the procerus muscle.

Each glabellar point was injected with 0.1 mL of a solution prepared by reconstituting 50 units of letibotulinumtoxinA in 1.25 mL of diluent, corresponding to 4 units per injection point, for a total of 20 units administered to the glabellar complex.

### 5.3. Injection Procedure

**Figure 3:**
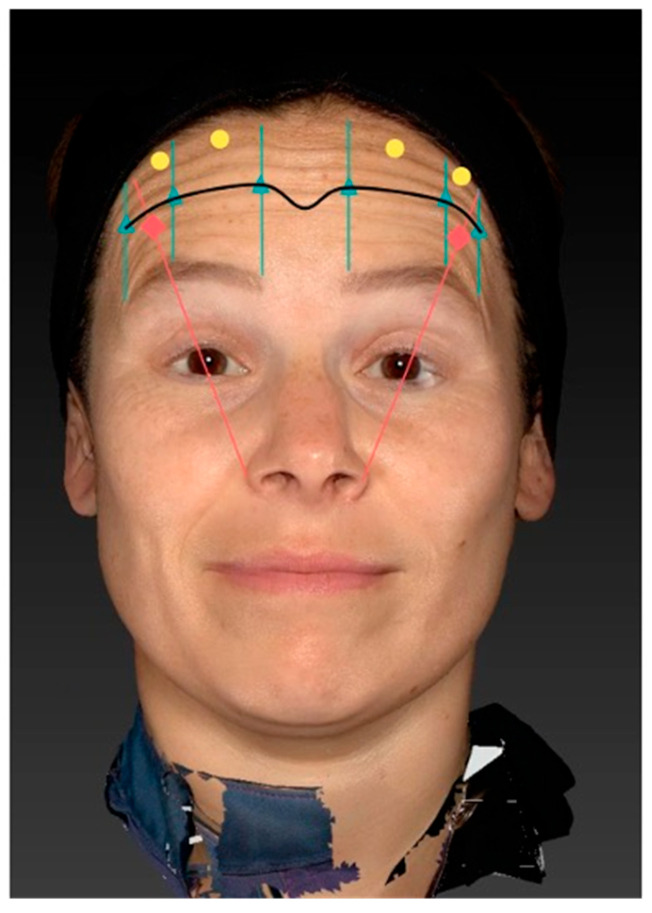
Illustration of the LEBO injection scheme for the frontalis muscle. The scheme integrates key anatomical landmarks, including the natural brow shape, the line of convergence (blue line), the lateral pull of the eyebrow, and areas of hyperactive muscular activity. The line of convergence is first identified by having the patient elevate the eyebrows while gently placing an index finger on the forehead to determine the point where cranial and caudal movement cancel out. Along this line, six deep intramuscular injections (yellow dots) of 2 units each are placed at the vertical intersections corresponding to the medial eyebrow, apex, and lateral eyebrow bilaterally. One superficial “balancing point” (green dot) is injected with 1 unit approximately 1.5–2 cm above the eyebrow to prevent excessive lateral pull and avoid the Mephisto sign. Additional “optimization points” (red dots) are placed intramuscularly with 1 unit each in regions of highest muscle activity, as determined individually.

The injection was performed after the patients had been marked for their injection points at the level of the C–line, their balancing points, and, if required, optimization points. After sterile disinfection with Chlorhexidine, the injection was performed using letibotulinumtoxinA (Croma^®^ Pharma GmbH, Leobendorf, Austria). 50U of letibotulinumtoxinA were reconstituted using 1.25 mL of NaCl. The reconstituted solution was drawn into 0.3 mL BD syringes (Becton Dickinson, Franklin Lakes, NJ, USA). The injection was performed at a 45° injection angle until the sub-frontalis fascia was pierced for the deep injection points, while a 20° injection angle was utilized for the subcutaneous injections (Figure 3).

### 5.4. Determination of Forehead Line Severity

**Figure 4:**
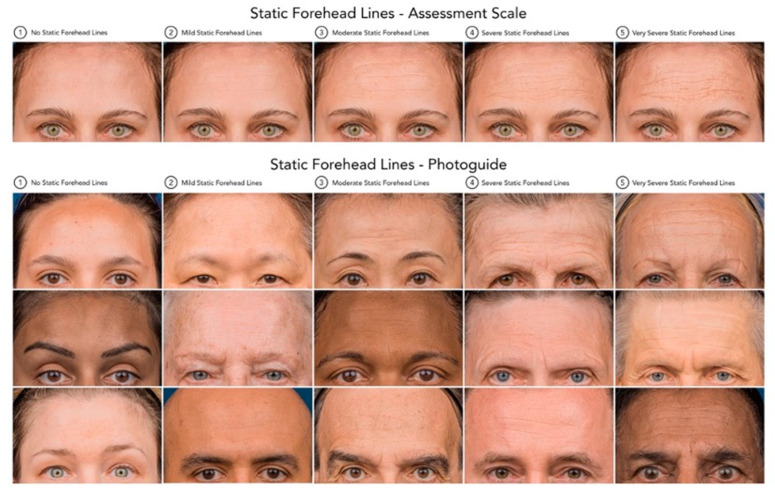
Static Forehead Lines—Assessment Scale and Photoguide. Standardized 5-point photonumeric scale used to assess the severity of static forehead lines in both live and digital evaluations. The upper panel illustrates the reference assessment scale ranging from (0) no static forehead lines to (4) very severe static forehead lines. The lower panel presents the corresponding validated photographic examples (Croma^®^ Static Forehead Lines Assessment Scale, CSFLAS) used as a visual guide to ensure consistency and reproducibility in rating static forehead line severity across subjects.

**Figure 5:**
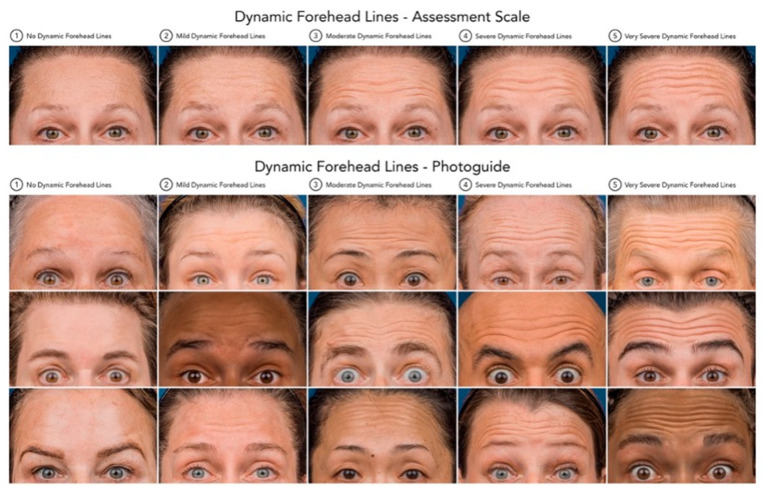
Dynamic Forehead Lines—Assessment Scale and Photoguide. Standardized 5-point photonumeric scale utilized to evaluate the severity of dynamic forehead lines during maximal frontalis contraction. The upper panel depicts the assessment scale ranging from (0) no dynamic forehead lines to (4) very severe dynamic forehead lines. The lower panel presents the corresponding validated photographic examples (Croma^®^ Dynamic Forehead Lines Assessment Scale, CDFLAS), serving as a reference tool to ensure consistent and objective grading of dynamic forehead line severity across clinical assessments and digital image analyses.

Forehead line severity was assessed both dynamically and at rest/statically. The assessment tool to rate the severity of the lines was the Croma^®^ Dynamic Forehead Lines Assessment Scale (CDFLAS) and the Croma^®^ Static Forehead Lines Assessment Scale (CSFLAS) (Figure 4 and Figure 5). These validated scales are 5-point photonumeric scales, which have been previously validated for their use in the live and digital setting. The assessment of forehead line severity was performed on the 3-Dimensional surface images taken using the Vectra H2^®^ (Canfield Scientific Inc. ^®^, Fairfield, NJ, USA) by the same rater (K.F.). The forehead line severity was assessed at baseline and after 2 weeks.

### 5.5. Dimensional Surface Imaging

Three-dimensional surface imaging was performed using the Vectra H2^®^ system, capturing standardized images of each subject at rest and during maximal eyebrow elevation. The images were then superimposed using the Vectra Software Suite^®^ 5.4 (Canfield Scientific Inc.^®^, Parsippany, NJ, USA). The software’s vector-tracking algorithm automatically identifies corresponding surface landmarks across both image sets and computes the displacement of each point in three dimensions. This algorithm quantifies skin movement by calculating vector magnitude (in millimeters) and direction across the forehead surface, producing an objective and reproducible map of regional motion. All analyses were performed retrospectively by the same investigator (K.F.) to ensure methodological consistency and minimize inter-observer variability.

### 5.6. Statistical Analysis

The data were analyzed using descriptive and inferential statistical methods to evaluate the effectiveness of the novel injection pattern. Continuous variables, such as age, dosage, and vertical skin displacement, were summarized using mean ± standard deviation (SD), while categorical variables, such as dynamic and static forehead line severity, were presented as frequencies and percentages. Proportions of patients achieving ≥1- or ≥2-point improvements were calculated to illustrate treatment effectiveness. All continuous variables were assessed for normal distribution using the Shapiro–Wilk test, and the variables analyzed (e.g., vertical skin displacement measurements) demonstrated normal distribution, supporting the use of parametric testing. For comparisons of continuous variables before and after treatment—such as vertical displacement at baseline versus follow-up—paired two-tailed *t*-tests were employed, selected based on both the normal distribution of the data and the paired study design. *p*-values were calculated directly from the t-statistics generated during these analyses, with statistical significance predefined as *p* < 0.05. All statistical analyses were performed using SPSS version 26.0 (IBM Corp., Armonk, NY, USA), and unless otherwise specified, data are reported as mean ± SD. 

## Figures and Tables

**Table 1:** Table showing the baseline demographics and the amount of toxin used for the injections. U = International Units.

ID	Age (Years)	Gender	Dosage Lateralization Points (U)	Dosage Balancing Points (U)	Number of Optimization Points	Units for Optimization Points (Total U)
1	46	F	12	2	2	2
2	37	M	12	2	2	2
3	44	F	12	2	2	2
4	49	M	12	2	4	4
5	37	M	12	2	4	4
6	41	F	12	2	4	4
7	45	F	12	2	4	4
8	30	F	12	2	4	4
9	47	F	12	2	4	4
10	27	M	12	2	4	4
11	35	M	12	2	4	4
12	55	F	12	2	4	4
13	36	M	12	2	4	4
14	39	F	12	2	4	4
15	40	M	12	2	4	4
16	37	F	12	2	4	4
17	54	M	12	2	4	4
18	48	F	12	2	4	4
19	66	F	12	2	4	4
20	40	F	12	2	4	4
21	47	M	12	2	4	4
22	40	M	12	2	4	4
23	50	F	12	2	4	4
24	30	F	12	2	4	4

## Data Availability

The raw data supporting the conclusions of this article will be made available by the authors on request. The data are not publicly available due to privacy and ethical restrictions, as they contain information that could compromise the confidentiality of research participants.
